# Availability and Affordability of Primary Health Care Among Vulnerable Populations in Urban Kumasi Metropolis: Family Health Perspective

**DOI:** 10.1089/heq.2021.0045

**Published:** 2022-05-11

**Authors:** Gertrude Acquah-Hagan, Daniel Boateng, Emmanuel Appiah-Brempong, Peter Twum, Joseph Amankwa Atta, Peter Agyei-Baffour

**Affiliations:** ^1^ Department of Health Policy, Management and Economics, School of Public Health, Kwame Nkrumah University of Science and Technology, Kumasi, Ghana.; ^2^ Suntreso Government Hospital, Kumasi, Ghana.; ^3^ Department of Epidemiology and Biostatistics, School of Public Health, Kwame Nkrumah University of Science and Technology, Kumasi.; ^4^ Department of Health Promotion and Disability Studies, School of Public Health, Kwame Nkrumah University of Science and Technology, Kumasi.; ^5^ Methodist Hospital Ankaase, Ashanti, Ghana.

**Keywords:** adequacy, affordability, availability, Ghana, quality of care, vulnerable populations

## Abstract

**Purpose:**

Health-related expenditures pose a significant burden on vulnerable populations. This study assessed the availability and affordability of primary health care among disadvantaged populations in urban Kumasi Metropolis, Ghana.

**Methods:**

This study was a descriptive cross-sectional study conducted among multi-level participants of vulnerable populations ≥18 years of age (*n*=710) constituting the older adults/aged, pregnant women, head porters, sex workers, and other vulnerable groups (people with disabilities and the homeless). Data were collected using a semistructured questionnaire. Poisson regression with robust variance was used to assess the association between vulnerability and access to health care.

**Results:**

There were significant differences in the availability and adequacy of health care among the vulnerable groups studied. Distance to the source of care was >5 km for majority of the vulnerable groups and the average expenditure on a visit to the health facility was GH¢ 27.04 (∼US$ 5.55 as at January 2019). Challenges to health care among the vulnerable groups included monetary (37.9%), stigmatization (18.6%), and staff attitude (25.9%). Head porters and other vulnerable groups were less likely to view health care as affordable compared with older adults. The difference in the perception of health care affordability was, however, explained by sociodemographic characteristic and health care-related factors.

**Conclusion:**

Despite the introduction of a National Health Insurance Scheme in Ghana, this study highlights challenges in health care access among vulnerable populations independent of the type of vulnerability. This suggests the need for stakeholders to adopt other innovative care strategies that may have broader applicability for all populations.

## Introduction

Globally, vulnerable populations are faced with health care challenges, and have poorer health outcomes.^1–3^ These challenges are more pronounced among vulnerable populations from low- and middle-income countries (LMICs) where the health system is fraught with inadequacies and lack of resources. Even though no clear consensus regarding what constitutes vulnerability has been reached till date, the general understanding in discussions concerning vulnerable populations in the domain of health and health care is that there is a direct linkage between vulnerability and poor health.^4,5^ Vulnerable populations may be defined as those at a higher risk of attaining poor health status and limited access to basic or primary health care (PHC).

The equitable Universal Health Coverage (UHC) defines access as “the opportunity and freedom to use health services.” It analyses access from three perspectives: (1) the provision of accessible and necessary services (“depth”), (2) for the entire population (“breadth”), and (3) accommodating the “differential needs” and financial constraints of disadvantaged groups (“height”).^6^

Adequate health care reaches people in need when it is available, accessible, acceptable, and known to them.^7,8^ In Ghana, rural–urban or north–south migration results in an increasing number of working and street children in urban cities like Accra and Kumasi, many of who work as head porters. These head porters are faced with extremely poor working and living conditions, homeless people with little access to health care or sanitation facilities.^9^ The older adult is also vulnerable to economic risks because of lower lifetime earnings and weakened social ties. Not only are the older adults disconnected from formal sector social protection systems but they also face the burden of high health care expenditure.^9^

Clients’ perception of worth relative to total cost is of importance to the affordability of services. One of the barriers to PHC among vulnerable groups is unaffordable costs to households. The prevalence of chronic and complex conditions, economic disadvantages, and the costs associated with health care can combine to make health services unaffordable for most vulnerable groups. It is most directly linked with dimensions of individuals’ income.^10^

There are also indirect costs beside informal payments and direct cost of health services that discourage the poor and vulnerable from seeking treatment. These indirect or consumer costs include transportation costs, the opportunity cost of time for both the patients and caregivers to accompany the client to the facility, and expenses on food and lodging.^10,11^

There is increasing focus on both financial barriers to accessing care and the economic consequences of paying for health services. Patients from vulnerable households often do not seek care or may do so only when they have access to funds. Continuity of care where and when required will not be effective due to limited funds.^8,12^ In South Africa, the highly vulnerable households had limited source of income such that they depend on gifts from friends and family members, which substantially affects regular consultations of health care.^8^ The poorest and vulnerable households in low-income settings with less health insurance borrowed money or sold items to pay for health care.^13,14^ Noncommunicable diseases particularly impose serious financial burden on poor and vulnerable households in low-income countries.^8^

In response to the financial challenges associated with accessing health care particularly among vulnerable populations, developing countries, including Ghana, are increasingly adopting financing arrangements to protect individuals against out-of-pocket payment and ensure increase in utilization of health care services. The economic benefit of the use of health insurance is to reduce out-of-pocket payment for health care by providing financial risk protection and reducing vulnerability and poverty.^15,16^ This concept addresses health care challenges faced by poor people, especially the vulnerable and rural residents.^17^

The introduction of the National Health Insurance Scheme (NHIS) in Ghana became imminent due to the failure of many health funding mechanisms, to ensure financial accessibility and UHC to the population.^18^ The NHIS program introduced an exemption scheme to improve access to affordable health care services among the poor and vulnerable (people with no source of income or fixed place of residence, nor live or depend on a person who is employed and has a fixed place of residence). Pregnant women and beneficiaries of Livelihood Empowerment Against Poverty^19^ are also excepted. Despite these, there are still high unmet needs in health care access. Health-related expenses pose a significant burden on vulnerable patients in Ghanaian societies.

Health care services that are designed to address the needs of the general population might lack the flexibility and responsiveness to meet the special health care needs of vulnerable populations. In 2016, a qualitative study in Ghana found that, although women with disability are willing to receive institutional maternal health care, their disability often made it difficult for such women to travel to access skilled care, as well as gain access to unfriendly physical health infrastructure.^20^

Rural-to-urban migrant women working in the informal sector, such as Ghana's head porters (*kayayei*), may also experience additional challenges in accessing primary care services due to marginalization and vulnerability resulting from both their gender and migrant status.^21^ Head porters experience exclusion from the health system, risk of being uninsured, and poor health outcomes. Among the older adults, similar challenges have been reported. A recent qualitative study reported inadequate information from health workers regarding care of the older person, queuing frustrations, and financial burden as key challenges in accessing primary care services in Ghana.^22^ These evidence shows system- and individual-level challenges to health care services among vulnerable populations and the lack of responsiveness of the health system to meet these challenges.

Few available evidence have also been focused on individual vulnerable groups, and no study has been conducted to assess financial challenges to health care services among different vulnerable populations to ascertain the access differentials among these populations in a health insurance setting like Ghana. The objective of this study was to assess the availability and affordability of PHC among vulnerable groups in the vulnerable enclaves of urban Kumasi Metropolis, Ghana.

## Methods

### Study design and population

This was a descriptive cross-sectional study conducted in the Kumasi metropolis of Ghana. This study site selection was based on its cosmopolitan nature, which makes it likely to have various vulnerable groups with diverse socioeconomic backgrounds. The Metropolis is divided into 10 submetropolitan districts. It is also endowed with 189 health facilities, of which about 150 are managed by private individuals.

The target population for this study was multilevel participants from vulnerable groups in Kumasi Metropolis, who were selected based on certain characteristics: individuals with risk or multiple risk factors to health care access. Individuals 18 years of age and older, who were identified as vulnerable by the vulnerability profile, were enrolled into the study. The target groups selected from the vulnerability enclaves were sex workers, people with disabilities, the homeless, female porters, pregnant women, older women, and orphans.

### Sampling and sample size

The sample size is calculated based on a formula by Kirkwood and Sterne,^23^ assuming that an access preference rate of 65% among the vulnerable population is required for improved health care access. With a 95% confidence interval (CI) and a significance level of 0.05, a sample size of 235 participants per vulnerable group was estimated to include a design effect of 1.2 and a nonresponse rate of 15%. The final sample included 359 older adults/aged, 117 pregnant women, 86 head porters, 75 sex workers, and 73 other vulnerable groups (people with disabilities and the homeless).

The study used cluster sampling to select individual households who were stratified by vulnerability profile. Using vulnerability enclaves, the study first selected clusters of communities that have been identified as busy with and/or occupied by vulnerable groups. Except for the aged and pregnant women, who were sampled at clinics in health facilities, all other vulnerable groups were identified using snowballing sampling technique. Individual sex workers, head porters, people with disabilities, and the homeless were first identified and through discussion showed the areas where they usually conglomerate.

In the selected vulnerability enclaves, simple random sampling technique where inscription of “Yes” and “No” were made on papers to pick without replacement was used to enroll vulnerable populations into the study. Respondents who picked “Yes” and consented were enrolled. This process was repeated until the required sample size was arrived at.

### Data collection

Data for this study were collected using a semistructured questionnaire which solicited data on the sociodemographic information such as age, gender, marital status, religion, occupation, income level, education level, assets owned by participants, and type of residence. The wealth status of the respondents was classified into low, medium, and high. Using a Principal Component Analysis, relative household wealth was assessed to assign the indicator weights to household indicators of respondents. The household wealth indicators included house ownership, house plots, store or containers, vehicles, motorbike, bicycle, a firm, refrigerator, computer, and sowing machine.

For reliability and validity of the study conclusions, a 3-day training session was held for the research assistants by the principal investigator. The data collection tools were pre-tested on 60 households before the data collection. The study was explained to respondents and consent was duly sought before data collection. A written informed consent was explained to participants who consented to the study before their enrolment. The study was approved by the Committee for Human Research Publication and Ethics at Kwame Nkrumah University of Science and Technology/Komfo Anokye Teaching Hospital, Kumasi, Ghana.

### Data analysis

Data were first presented as frequency tables or charts to assess the distribution. The bivariate analysis involved the use of Pearson Chi-square or Fischer's exact test and one-way analysis of variance to assess association of the perception of service affordability and categorical and continuous variables, respectively. Poisson regression with robust variance was used to estimate prevalence ratios (PRs) and corresponding 95% CIs and *p*-values.^24^ The two-sided significance level was set at 0.05.

#### The Poisson regression with robust variance

The popular modified Poisson regression approach^24,25^ has been suggested to estimate relative risk in the form of PRs in cross-sectional studies where outcomes are not rare, >10%. This method also uses a log link and, hence, has the same formula as log binomial distribution:

(1) $$\log\left(\pi_{i}\right)=\beta_{0}+\beta_{1}\beta_{1i}+\beta_{2}\beta_{2i}+\ldots +\beta_{k}X_{ki}$$

where π*i* is the probability of experiencing the outcome of interest for subject *i*, and *X*_1_*i*, *X*_2_*i*,…, *X_ki_* are predictor variables.

However, the Poisson regression applies a Poisson distribution to the data, rather than a binomial distribution. It produces consistent estimates of the parameters in Equation 1, but inconsistent variances, since the variance under a Poisson model is larger than the variance under a binomial model unless the outcome is rare.^26^ Robust variance estimation is therefore used to avoid overestimating standard errors of parameter estimates.^24^ The modified Poisson regression approach has been applied across a broad range of observational^27,28^ and intervention studies.^29,30^

The outcome in this study was the perception of health care affordability, coded as “1=affordable” and “0=not affordable.” The key explanatory variable, *X*_1_*i*, was type of vulnerable group: *older adults/aged, pregnant women, head porters, sex workers, and other vulnerable groups*. The older adults/aged was used as the reference group in the regression analysis.

Other predictor variables were used as control variables to adjust for confounding. This included age, education, employment status, marital status, religion, residence, relationship with health care providers, and source of health care payment. Two multivariable models were fitted to adjust for confounding variables. Model 1 adjusted for age, education, employment status, marital status, religion, and residence, and model 2 further adjusted for relationship with health care providers and source of health care payment.

## Results

### Background characteristics of respondents

Table 1 presents the sociodemographic characteristics of the vulnerable groups which were significantly different. Almost three-fourths of the respondents (74.6%) were females, and the mean (standard deviation) age of the participants was 51 (21.0) years. About a third of the participants had no formal education and only 23.4% and 5.4% had Senior High School and tertiary education, respectively. The proportion of respondents with tertiary education was highest among pregnant women (10.2%), whereas none of the ‘other vulnerable group’ category had tertiary education.

**Table 1.** Background Characteristics of Vulnerable Groups

Background characteristics	Vulnerable groups						***p***
	Total sample, ***n*** (%)	Older adults, %	Pregnant women, %	Female potters, %	Sex worker, %	Other,^a^ %	
Gender							
Male	180 (25.4)	36.8	—	—	—	11.8	
Female	530 (74.6)	63.2	100.0	100.0	100.0	88.2	
Age, mean (SEM)	51 (0.8)	68 (0.3)	30 (0.5)	27 (0.7)	28 (0.9)	20 (0.8)	<0.001
Level of education							<0.001
None	252 (35.5)	35.3	21.2	24.4	31.5	29.4	
Basic education (primary and JSS)	254 (35.7)	30.6	47.4	60.5	27.4	64.7	
Senior high school/middle school	166 (23.4)	29.4	21.2	12.8	32.9	5.9	
Tertiary/professional certificate	38 (5.4)	4.7	10.2	2.3	8.2	0.0	
Employment (*n*=674)							<0.001
Employed	438 (65.0)	44.0	85.4	95.3	96.0	52.9	
Unemployed	236 (35.0)	56.0	14.6	4.7	4.0	47.1	
Marital status (*n*=657)							<0.001
Single	103 (15.7)	2.9	11.9	19.8	74.7	30.8	
Married/co-habitation	353 (53.7)	43.4	84.4	80.2	13.3	69.2	
Divorced/widowed	201 (30.6)	53.7	3.7	0.0	12.0	0.0	
Number of children (*n*=587), mean (SEM)	4 (0.1)	5 (0.1)	2.5 (0.1)	2 (0.2)	1.6 (0.1)	1.5 (0.3)	<0.001
Ethnic group (*n*=666)							<0.001
Akan	512 (72.5)	91.6	67.2	9.3	58.7	79.2	
Northerner	136 (19.2)	6.1	20.4	82.6	5.3	20.8	
Other	59 (8.3)	2.2	12.4	8.1	36.0	0.0	
Religion (*n*=671)							<0.001
Christian	573 (85.4)	96.6	86.1	38.8	94.7	41.2	
Muslim	90 (13.4)	2.5	13.9	58.8	2.7	58.8	
Other	10 (14.9)	0.8	0.0	2.4	2.7	0.0	
Average monthly income *in GH¢* (mean, SEM)	399.04 (16.8)	467.90 (27.6)	266.26 (20.5)	380.94 (24.9)	470.43 (65.1)	132.60 (34.6)	<0.001
Place of resident (*n*=653)							<0.001
Zongo/old town	337 (51.6)	54.2	60.3	37.2	33.5	87.5	
Slum	86 (13.2)	3.5	13.0	55.8	9.3	12.5	
New site	178 (27.3)	37.7	17.6	3.5	29.3	0.0	
Estate/other	52 (8.0)	4.6	9.2	3.5	28.0	0.0	
Wealth quintiles							<0.001
Lowest	145 (22.4)	19.9	14.9	37.7	23.3	56.3	
Low	115 (17.7)	10.5	33.6	15.7	21.9	31.3	
Medium	144 (22.2)	26.6	17.9	15.7	20.5	6.3	
High	125 (19.3)	28.9	11.2	4.8	9.6	0.0	
Highest	119 (18.4)	14.0	22.4	26.5	24.7	6.3	

^a^ People with disabilities, drug users, prisoners, orphans.

JSS, Junior Secondary School; SEM, standard error of the mean.

About 35% of the respondents were unemployed, with significant differences among the vulnerable groups. The proportion of unemployment was highest among the older adults (56%) and lowest among the sex workers (4%). Majority of respondents belonged to the lower and low wealth quintiles.

### Availability and adequacy of PHC among vulnerable groups

Table 2 shows results of the availability and adequacy of PHC among various vulnerable groups. Availability of health professionals at the preferred health facility was not different among the vulnerable groups. Very few of the respondents from all groups described their relationship with health professionals as bad, except for other vulnerable groups, where 22% described their relationship with health professionals as bad. Attitude of health professionals was mostly described as positive by most of the vulnerable groups. The proportion who viewed health attitudes of health professionals as very positive was higher among the older adults (36.6%). Satisfaction with adequacy of health services received also differed significantly among the vulnerable groups (*p*<0.001).

**Table 2.** Availability and Adequacy of Primary Health Care Among Vulnerable Groups

Variables	Total sample, %	Vulnerable groups					***p***
		Older adults, %	Pregnant women, %	Female potters, %	Sex workers, %	Other,^a^ %	
Health professionals easily available when you visit preferred health facility	93.0	95.5	97.0	92.9	90.4	94.0	0.269
How do the professionals relate to you?							<0.001
Cordial	37.6	46.5	37.6	30.6	23.3	8.0	
Good	57.6	51.3	59.4	60.0	74.0	70.0	
Bad	4.7	2.3	3.0	9.4	2.7	22.0	
Attitude of health professionals at your preferred health facility							<0.001
Very negative	0.7	1.4	0.0	0.0	0.0	0.0	
Negative	1.6	0.8	1.5	7.2	0.0	0.0	
Neutral	12.3	4.8	17.4	18.1	16.4	38.3	
Positive	62.9	56.3	72.7	60.2	80.8	61.7	
Very positive	22.5	36.6	8.3	14.5	2.7	0.0	
Ever been referred by providers	11.7	8.6	15.7	18.8	13.9	14.0	0.035
Physical location of the facility easily reachable	93.1	95.2	95.5	92.9	100.0	88.0	0.044
Service provider has enough time for you when you visit	90.0	95.2	95.5	87.1	89.0	70.0	<0.001
Satisfaction of adequacy of services received in the last 12 months							<0.001
Dissatisfied	3.7	4.5	0.0	4.7	4.1	0.0	
Unsure	16.9	16.6	13.2	18.8	5.5	42.0	
Satisfied	68.6	68.5	69.8	61.2	84.9	56.0	
Very satisfied	10.7	10.4	17.1	15.3	5.5	2.0	

^a^ People with disabilities, drug users, prisoners, orphans.

### Perception of health care adequacy and availability

Table 3 shows results of the perceptions of the adequacy of PHC among the vulnerable groups studied. The health facility was the usual source of health care for most of the respondents. Among the sex workers, majority disclosed that they are seen by the same health staff when they visit the health facility, whereas majority of the other vulnerable groups disclosed they are not seen by the same health staff (*p*<0.001).

**Table 3.** Appraisal of the Concept of Primary Health Care from the View of Vulnerable Groups

Variables	Total sample, %	Vulnerable groups					***p***
		Older adults, %	Pregnant women, %	Female potters, %	Sex workers, %	Other,^a^ %	
Health facility usual source of health care (*n*=696)							<0.001
Yes	79.8	76.6	88.7	76.8	93.2	64.0	
No	20.2	23.4	11.3	23.2	6.8	36.0	
Seen by the same health staff (*n*=696)							<0.001
Yes	30.0	23.4	25.2	39.8	60.3	30.0	
No	70.0	76.7	74.8	60.2	39.7	70.0	
Distance to source of care (km; *n*=657)							<0.001
<1	12.7	6.6	10.2	13.6	51.6	8.0	
1–3	10.3	11.5	13.4	9.9	4.7	2.0	
3–5	21.0	21.8	23.6	14.8	14.1	28.0	
>5	56.1	60.1	52.8	61.7	29.7	62.0	
Health staff available when you visit the health facility (*n*=653)	97.2	99.4	100.0	90.1	96.8	86.7	<0.001
Waiting time (hours; *n*=689)							<0.001
<1	27.9	11.2	38.9	48.8	60.0	34.0	
1–2	59.2	67.6	54.2	47.7	35.7	66.0	
>2	12.9	21.2	6.9	3.5	4.3	0.0	
Working hours at which health staff usually available (*n*=686)							0.045
Day hours	98.4	98.3	99.2	100.0	94.5	100.0	
Night hours	1.6	1.7	0.8	0.0	5.5	0.0	
Monthly visit to source of care							<0.001
0	3.7	0.8	8.0	11.6	2.7	0.0	
1	87.7	95.3	83.2	79.1	66.7	92.5	
2	4.6	3.3	7.3	8.1	0.0	7.5	
3 or more	3.9	0.6	1.5	1.2	30.7	0.0	
Average visit at your last sickness before seeing a health staff? (*n*=633)							<0.001
0	4.9	2.0	9.3	13.9	2.8	0.0	
1	81.4	74.6	83.7	81.0	95.8	97.8	
2	13.0	21.8	7.0	5.1	1.4	2.2	
3 or more	0.8	1.7	0.1	0.1	0.1	0.1	
Arrangements are made for services not rendered (*n*=222)	45.9	35.4	36.4	73.9	59.6	50.0	<0.001

^a^ People with disabilities, drug users, prisoners, orphans.

Distance to the source of care was >5 km for a majority of the vulnerable groups, except for sex workers, among whom the majority travelled <1 km to their source of care. Health staff were mostly available, during day time hours and a majority waited for between 1 and 2 h to be seen by a health staff. Most vulnerable groups saw a health staff after making one visit to the health facility. However, 21.8% and 7% of the older adults and pregnant women, respectively, saw a health staff on an average of two visits to the health facility. Out of 222 respondents, more than half disclosed that no arrangement was made for services that were not rendered to them at the health facility.

As shown in Table 4, the average transport cost to the health facility was highest among sex workers (GH¢ 8.14) and lowest among other vulnerable groups (GH¢ 2.71). Average monthly expenditure when they visit their source of care was also highest among sex workers and lowest among pregnant women. The proportion who viewed their monthly expenditure as affordable was highest among pregnant women (81.0%) and lowest among other vulnerable groups (24.4%), among whom majority found their monthly expenditure as not affordable. Source of payment for health care was significantly different among the vulnerable groups. Almost half of the sex workers, compared to less than a quarter of other vulnerable groups, paid for their health care themselves.

**Table 4.** Service Affordability of Primary Health Care Among Vulnerable Groups

Service affordability	Total sample, %	Vulnerable groups					***p***
		Older adults, %	Pregnant women, %	Female potters, %	Sex workers, %	Other,^a^ %	
Average transport cost (GH¢), mean (SEM)	3.80 (0.2)	3.68 (0.2)	3.06 (0.2)	2.86 (0.2)	8.14 (1.0)	2.71 (0.2)	<0.001
Average expenditure when you visit your source of care (GH¢), mean (SEM)	27.04 (1.6)	23.59 (1.7)	18.32 (2.5)	21.13 (3.4)	59.46 (7.1)	20.9 (3.1)	<0.001
Average monthly expenditure on health care (GH¢), mean (SEM)	37.20 (1.9)	34.84 (2.6)	28.77 (3.8)	34.57 (6.0)	65.27 (7.3)	32.89 (5.1)	<0.001
View about this monthly expenditure							<0.001
Affordable	60.6	64.6	81.0	50.0	73.5	24.4	
Not affordable	26.1	25.9	16.2	28.6	20.8	55.6	
Reasonable	13.3	9.5	2.9	21.4	5.6	20.0	
Source of payment for your health care							<0.001
Self-financing	19.7	18.8	19.0	20.3	50.7	20.8	
NHIS subscription	79.4	80.5	81.0	77.9	49.3	79.2	
Family members	0.6	0.8	0	0	0	0	
Friends	0.3	0.0	0.0	2.3	0.0	0.0	
Sources of payment able to get all medications prescribed	70.9	70.0	74.8	75.3	60.3	75.6	0.175
Anyone accompanies you to the source of care	26.9	37.7	11.3	12.9	32.9	6.0	<0.001
Pay for the services of the caregiver	18.7	4.7	29.7	33.3	8.2	0.0	<0.001

^a^ People with disabilities, drug users, prisoners, orphans.

NHIS, National Health Insurance Scheme.

**FIG. 1.**
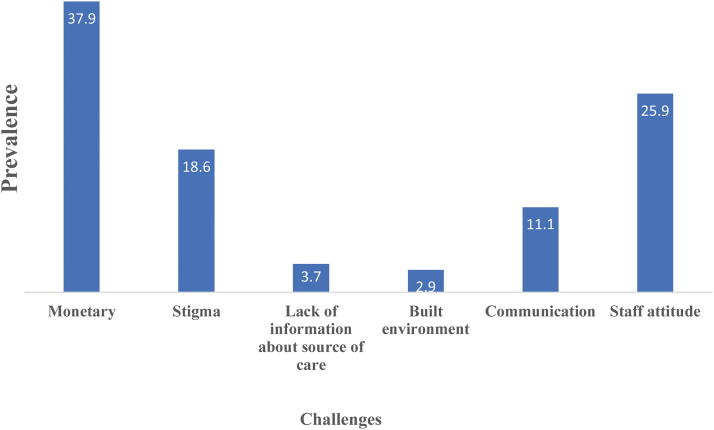
Challenges faced by vulnerable populations in seeking health care.

Almost 38% of vulnerable populations studied disclosed that monetary issue was a challenge to seeking health care (Fig. 1). Other challenges mentioned included stigmatization (18.6%), communication with health staff (11.1%), and staff attitude (25.9%).

### Association between type of vulnerable group and perception of health care affordability

In the crude models, pregnant women had lower PR of perception of health care affordability compared to older adults (PR: 0.88, 95% CI: 0.80–0.98), Table 5. Head porters and other vulnerable groups were also less likely to view health care as affordable compared to older adults. The differences in health care access observed were, however, attenuated after adjustment for sociodemographic characteristics and health care-related factors. Further analysis stratified by sex showed differential associations between sociodemographic and health care-related factors.

**Table 5.** Prevalence Ratios and 95% Confidence Interval for the Association Between Type of Vulnerability and Affordability of Health Care

Covariates	Affordability of health care					
	PR [95% CI]	***p***	Model 1, PR [95% CI]	***p***	Model 2, PR [95% CI]	***p***
Vulnerable groups						
Older adults	1.00		1.00		1.00	
Pregnant women	0.94 [0.80–1.12]	0.513	0.97 [0.58–1.65]	0.334	0.84 [0.51–1.37]	0.424
Head porters	0.78 [0.61–0.99]	0.043	0.92 [0.50–1.71]	0.941	0.92 [0.53–1.58]	0.648
Sex workers	1.14 [0.97–1.34]	0.105	1.06 [0.56–1.99]	0.480	1.17 [0.69–2.00]	0.208
Others	0.38 [0.23–0.64]	<0.001	1.23 [0.46–3.30]	0.452	1.52 [0.60–3.90]	0.821

Model 1: Adjusted for age, education, employment status, marital status, religion, residence. Model 2: Model 1 + relationship with health care providers, source of health care payment.

CI, confidence interval; PR, prevalence ratio.

Being in the high wealth quintile was associated with a lower prevalence of perceiving health care as affordable among the older adults, whereas having a medium health quintile was associated with a lower prevalence of perceiving health care as affordable among other vulnerable populations. Having a good relationship with health staff was also positively associated with perception of health care affordability among older adults and other vulnerable groups as shown in Supplementary Table S1.

## Discussion

The findings of this study show differences in the availability and adequacy of PHC as well as perception of health care affordability among the vulnerable groups. The NHIS was the main source of payment for health care among vulnerable groups. Some older adults, pregnant women, sex workers, head porters, and a majority of other vulnerable groups viewed health care as not affordable. The differences in the perception of health care affordability could be explained by the sociodemographic and health care-related characteristics of vulnerable groups.

Among the vulnerable groups studied, majority travelled 5 km or more to the health facility. In low-income settings, long distance to health facilities excludes people from accessing health care and this is reported to be more challenging among vulnerable populations such as pregnant women, the older adults, and the disabled.^31^ Among pregnant women, distance to health facilities influence antenatal and post-natal care utilization and access to skilled delivery in both urban and rural settings.^32,33^

Among older adults, distance to health facilities is a major barrier to accessing health care even in the advent of exemptions from cost of health care and consultation fees.^34–36^ Geographic distance has also been reported as a major access barrier among people with disability, which was worsened by transportation problems.^37^ It is eminent to assess proximity of health care services as well as barriers to movement to enhance access to health care services, especially among vulnerable populations.^38^ Technological provisions such as Geographical Information Systems have for instance been employed to quantify the health needs of people by analyzing health care accessibility among groups.^39^

Financial challenge was a major reason for not having optimal access to health care among vulnerable groups in this study. The average monthly health care expenditure was highest among sex workers and lowest among pregnant women. Despite the impact of the NHIS on Out-of-Pocket Expenditures over the last 14 years, health care cost is still found to be catastrophic for a large proportion of insured households in Ghana.^40^ People with limited financial resources have challenges in accessing health care. This is consistent with previous studies that found financial challenges as major barriers to health care access among the poor and vulnerable populations.^2,10,41^ Financial barriers to health care among vulnerable populations relate to their inability to pay for consultations, medications, and cost of transport to the health facilities. Overall, poor older adults have been found to use health services much less than the nonpoor older adults even when they are enrolled on the NHIS.^42^

Source of payment for health care was significantly different among vulnerable groups, with almost half the sex workers paying for their health care expenditure compared to less than a quarter of the other vulnerable groups, most of whom relied on the NHIS. Cost of health care was viewed as unaffordable for some vulnerable groups. This suggests that the NHIS is unable to fully cater for cost of health care for vulnerable groups.^43,44^ As suggested by Alfers,^45^ apart from their inability to pay for the NHIS premium, most head porters, for instance, complain that the scheme does not work when used in accessing health care.

The working and living conditions of these vulnerable groups also expose them further to health problems, increasing their expenditure on health care.^37,46,47^ Socioeconomic characteristics explained the differences in the perception of health care affordability among the vulnerable groups studied. There was a generally low socioeconomic status among vulnerable populations. The income levels of study participants were very low; the average monthly income was GH¢ 399.04 (∼US$ 81.30), which was just a little above the minimum wage as at January 2019 GH¢ 319.50 (∼US$ 58.20) as of January 2019. More than 60% were in the moderate to lowest wealth quintile.

Although the influence of perception of the quality of health care is well documented,^48,49^ how this is expressed among vulnerable populations has not been given much attention. Attitude of health staff was a challenge to accessing health care among the vulnerable groups. Although majority of respondents disclosed a positive association with health staff, some stated otherwise and the relationship with health staff was associated with the perception of health care affordability. Perceived nonrespectful attitude and unapproachable interaction style of formal health care providers were reported as barriers to formal health care utilization in Ghana.^50^ Older people disclosed their disappointment with health provision as their expectations are always not met, thereby deciding to stay away from utilizing formal health care.^50^ The perceived poor attitude of health workers and its influence on health care utilization among older people have also been reported in previous research in both high-^35^ and low-income settings.^34,51^

### Implications of findings

Vulnerable pupations, especially from LMICs, are faced with challenges in accessing health care. This is further exacerbated by the increasing cost of health care due to increasing prevalence of chronic and complex conditions and economic disadvantages. Despite the introduction of financial arrangements such as the NHIS in response to the financial challenges associated with accessing health care, vulnerable populations still face high unmet needs in health care access.

This study supports the call for strengthening and monitoring of interventional programs aimed at supporting vulnerable populations to access health care. For instance, the policy on exemption under the NHIS needs to be revised and further strengthened to ensure that it serves the populations and purpose it intends to serve. The differences in the ability to afford health care among various vulnerable groups might depend on their socioeconomic status and ability to pay for health care. Vulnerability-friendly organizations should also advocate for more governmental support to economically empower vulnerable populations.

### Strengths and limitations of the study

This is the first study to explore the availability and affordability of health care among various vulnerable groups in Ghana. This study utilized consistent measures and data collection across sites, to enhance the generalizability and applicability of the study finding to the setting. A limitation of the study is the use of cross-sectional design and inability make causal inference on the relationship between affordability of PHC and vulnerable groups.

There may be an issue of potential endogeneity from this study, given the descriptive feature. Some of the explanatory variables may be driven by other variables that were not considered or explored in this study. For example, there may exist some variables that affect both affordability of PHC and vulnerable groups, but were not taken into consideration in the analysis. Econometric techniques such as the use of instrumental-variable regression could be explored on a relatively larger sample size to control for this.^52^

The study might also suffer from respondents and social desirability bias, respondents might have the propensity to give more acceptable responses than what is more reflective of their thoughts about the availability and affordability of health care.^53^ These notwithstanding, we belief that the reliability and validity measures upheld in this study were protective for the policy utilities of our findings.

## Conclusion

In conclusion, this study has also demonstrated the need for support for vulnerable populations to be able to access available primary health services. Financial challenges, long distances, and attitude of health staff influenced access to health care among vulnerable populations. Although there has been an implementation of the NHIS with focus on vulnerable populations, this has not been able to fully alleviate financial challenges to health care access among vulnerable populations. This suggests the need for stakeholders to adopt other innovative care strategies that may have broader applicability for all populations.

## Supplementary Material

Supplemental data

## Data Availability

The datasets used and/or analyzed during this study are available from the corresponding author on reasonable request.

## Abbreviations Used

CI: confidence interval
LMICs: low- and middle-income countries
NHIS: National Health Insurance Scheme
PHC: primary health care
PR: prevalence ratio
SEM: standard error of the mean
UHC: Universal Health Coverage
