# Supplementary material for: Availability and Affordability of Primary Health Care Among Vulnerable Populations in Urban Kumasi Metropolis: Family Health Perspective
Source: Health Equity. 2022 May 11;6(1):345–55. doi: 10.1089/heq.2021.0045 (PMC9148648; doi:10.1089/heq.2021.0045)
Supplement: Supplemental data [file Suppl_TableS1.docx]

**Supplementary Table 1.** Prevalence ratios and 95% CI for the association between socio-economic factors, affordability of healthcare and vulnerable groups

| **Covariates** | **Affordability of healthcare** | | |
| --- | --- | --- | --- |
|  | **Elderly**  **PR [95% CI]** | **Pregnant women**  **PR [95% CI]** | **Others***†*  **PR [95% CI]** |
| **Age** | 1.00 [0.98, 1.02] | 1.01 [0.98, 1.05] | 1.03 [1.01, 1.05]** |
| **Level of education**   - None - Basic - Senior High School - Tertiary | 1.00  0.68 [0.50, 0.93]*  1.00 [0.75, 1.33]  0.78 [0.39, 1.55] | 1.00  1.12 [0.64, 1.98]  1.38 [0.76, 2.53]  1.66 [0.61, 4.54] | 1.00  1.03 [0.72, 1.46]  0.95 [0.70, 1.27]  1.13 [0.73, 1.75] |
| **Unemployed** | 1.10 [0.86, 1.40] | 0.95 [0.48, 1.91] | 0.99 [0.66, 1.49] |
| **Marital status**   - Single - Married/ Co-habitation - Divorced/ Widow | 1.00  1.54 [0.59, 3.98]  2.04 [0.81, 5.16] | 1.00  0.73 [0.38, 1.43]  0.56 [1.18, 1.58] | 1.00  0.45 [0.31, 0.64]***  0.75 [0.40, 1.40] |
| **Religion**   - Christian - Muslim - Other | 1.00  0.84 [0.42, 1.67]  ( - ) | 1.00  1.35 [0.76, 2.40]  (-) | 1.00  0.92 [0.57, 1.48]  1.36 [0.90, 2.07] |
| **Wealth quintiles**   - Low - Medium - High | 1.00  0.88 [0.66, 1.18]  0.61 [0.44, 0.86]** | 1.00  1.34 [0.76, 2.38]  1.02 [0.64, 1.63] | 1.00  0.53 [0.33, 0.84]**  0.79 [0.58, 1.08] |
| **Residence**   - Zongo/ old town - Slum - New site - Estate/ other | 1.00  1.52 [1.20, 1.93]***  2.00 [1.50, 2.68]***  1.40 [0.73, 2.71] | 1.00  1.55 [1.03, 2.36]*  0.89 [0.50, 1.59]  0.44 [0.08, 2.34] | 1.00  1.00 [0.65, 1.54]  0.70 [0.45, 1.08]  0.92 [0.65, 1.30] |
| ***Healthcare related factors*** |  |  |  |
| Pay healthcare through NHIS | 5.15 [2.66, 9.97]*** | 0.83 [0.59, 1.17] | 0.81 [0.61, 1.07] |
| Good relationship with health staff | 1.26 [1.09, 1.47]** | 1.14 [0.80, 1.54] | 1.63 [1.27, 2.09]*** |

**P<0.05; **p<0.01; ***p<0.001; PR=Prevalence ratios*

*†Include Sex workers, Head Potters, Disabled, street participants*
